# Effects of combined cannabidiol (CBD) and hops (*Humulus lupulus*) terpene extract treatment on RAW 264.7 macrophage viability and inflammatory markers

**DOI:** 10.1007/s13659-023-00382-3

**Published:** 2023-06-07

**Authors:** Inga Dammann, Claudia Keil, Iris Hardewig, Elżbieta Skrzydlewska, Michał Biernacki, Hajo Haase

**Affiliations:** 1 Sanity Group GmbH, Jägerstraße 28-31, 10117 Berlin, Germany; 2 Department of Food Chemistry and Toxicology, Technische Universität Berlin, Straße Des 17. Juni 135, 10623 Berlin, Germany; 3 Department of Analytical Chemistry, Medical University of Bialystok, A. Mickiewicza 2D, 15-222 Bialystok, Poland

**Keywords:** CBD, Terpenes, Cannabinoids, Entourage effect, Inflammation

## Abstract

This study investigates the potential of cannabidiol (CBD), one major cannabinoid of the plant *Cannabis sativa*, alone and in combination with a terpene-enriched extract from *Humulus lupulus* (“Hops 1”), on the LPS-response of RAW 264.7 macrophages as an established in vitro model of inflammation. With the present study, we could support earlier findings of the anti-inflammatory potential of CBD, which showed a dose-dependent [0–5 µM] reduction in nitric oxide and tumor necrosis factor-alpha (TNF-α) released by LPS-stimulated RAW 264.7 macrophages. Moreover, we observed an additive anti-inflammatory effect after combined CBD [5 µM] and hops extract [40 µg/mL] treatment. The combination of CBD and Hops 1 showed effects in LPS-stimulated RAW 264.7 cells superior to the single substance treatments and akin to the control hydrocortisone. Furthermore, cellular CBD uptake increased dose-dependently in the presence of terpenes from Hops 1 extract. The anti-inflammatory effect of CBD and its cellular uptake positively correlated with terpene concentration, as indicated by comparison with a hemp extract containing both CBD and terpenes. These findings may contribute to the postulations for the so-called “entourage effect” between cannabinoids and terpenes and support the potential of CBD combined with phytomolecules from a non-cannabinoid source, such as hops, for the treatment of inflammatory diseases.

**Supplementary Information:**

The online version contains supplementary material available at 10.1007/s13659-023-00382-3.

## Introduction

The standard treatment for acute and chronic inflammatory diseases mainly consists of corticosteroid-based over-the-counter formulations and prescriptions aimed at alleviating inflammatory symptoms and suppressing inflammatory signaling. However, the long-term systemic administration of corticosteroids poses a considerable risk of severe side effects including osteoporosis, hyperlipidemia, growth suppressions, gastrointestinal and hepatic effects, skin thinning, and increased risk for skin infections [1–3]. Therefore, the objective of this study was to evaluate the potential of a combined CBD-hops extract treatment as an alternative therapy option for various inflammatory diseases in an in vitro model of inflammation.

Cannabinoids are a group of lipophilic compounds that can be produced by the human body (“endocannabinoids”) and are also present in the plant *Cannabis sativa* (“phytocannabinoids”). They offer a promising alternative to the use of more aggressive treatments with common corticosteroids, as they modulate various biochemical pathways in the body, including those involved in inflammatory processes [4–6]. Notably, the non-psychotropic phytocannabinoid cannabidiol (CBD) shows promising potential as an alternative treatment option, due to its high abundance in the cannabis plant, its extensive anti-inflammatory and anti-oxidative potency, and little to no side effects observed from either topical or systemic administration [7–12]. Several in vitro and ex vivo studies have demonstrated that CBD possesses anti-inflammatory properties. Mechanistically, this is due to its modulation of the TLR4 signal transduction cascade and altered activation of the transcription factors NF-κB (nuclear factor κ-light-chain-enhancer of activated B cells) or AP-1 (activating protein-1), leading to lower levels of pro-inflammatory cytokines, such as tumor necrosis factor (TNF)-α [13–16].

The interaction of both endo- and phytocannabinoids with cellular receptors in the human body has uncovered a highly complex network comprising cellular receptors, lipid signaling molecules, and metabolizing enzymes, known as the “endocannabinoid system” (ECS) [17]. Initially, it was believed that the ECS was limited to the central and peripheral nervous system, but it is now known to extend to various organs in the human body, including the skin and the gastrointestinal system [18–20]. Disruptions in this intricate and finely balanced system could contribute to the development of pathological inflammatory conditions [17, 21].

Besides phytocannabinoids, the cannabis plant is known to contain numerous secondary plant metabolites, such as polyphenols and terpenes [22, 23]. The potential synergistic effect of these individual cannabis constituents acting together and exhibiting a stronger pharmacological potential than the added effects observed with the single components is commonly referred to as the “entourage effect” [24, 25]. Despite extensive speculation regarding the entourage effect in recent years, scientific evidence remains controversial [26–29]. This study not only focuses on evaluating the suitability of cannabidiol as a novel treatment method for inflammatory diseases with minimal known adverse effects but also addresses the possible entourage effect between cannabinoids and secondary metabolites naturally occurring in the cannabis plant (i.e., terpenes), with the aim of augmenting the known anti-inflammatory effect of cannabidiol [30]. *H. lupulus* is closely related to *C. sativa*, as both belong to the botanical family of Cannabaceae [31]. Despite this similarity, however, little research was done on the interrelation between plant constituents of both origins [31]. We propose that the unique combination of synthetic cannabidiol and extracts from hops is superior to regular extracts from *C. sativa*, which naturally contain terpenes as secondary metabolites. Thus, the risk of residing trace amounts of the psychotropic and strictly regulated cannabinoid ∆9-tetrahydrocannabinol (THC) [32] can be avoided by combining synthetic CBD and terpene-enriched extracts from *H. lupulus*, which is naturally devoid of cannabinoids [31, 33]. In this study, we aim to investigate the entourage effect between cannabidiol and phytomolecule assemblages present in extracts from either *C. sativa* or *H. lupulus*. Specifically, we elaborate on their respective benefits by evaluating cell viability, anti-inflammatory properties, and CBD uptake in lipopolysaccharide-stimulated RAW 264.7 mouse macrophages.

## Results

The RAW 264.7 mouse macrophage cell line is widely used to investigate anti-inflammatory effects of drugs and to assess associated signaling pathways, including those dysregulated in various inflammatory conditions [34–36]. Pro-inflammatory signaling by the Toll-like-4 receptor (TLR4) cascades [37] can be activated in these cells by stimuli such as bacterial lipopolysaccharides (LPS). This leads to increased production of nitric oxide (NO·) and pro-inflammatory cytokines, which serve as markers to verify the inflammatory activation of the cells [38, 39]. Therefore, the RAW 264.7 cell line was selected for in vitro investigations on the anti-inflammatory effects of pure CBD and standardized extracts of *H. lupulus*, as well as possible interactive effects when administered in combination, mimicking the phytocannabinoid‐terpenoid entourage effect that is postulated in cannabis research [40, 41]. Hydrocortisone was used as a positive control as it is known to attenuate the mRNA expression of proinflammatory cytokines and the gene expression of inducible nitric oxide synthase (iNOS) following LPS stimulation of RAW 264.7 cells [38]. The study also compared the combination of synthetic CBD and terpenes from hops extracts to extracts from *C. sativa*, which naturally contain both cannabinoids and terpenes. Following the approach of previous in vitro cannabidiol studies[42, 43], we decided to investigate the effect of CBD in combination with hops extracts on cell viability of RAW 264.7 and inflammatory markers (NO·, TNF-α) after stimulation with LPS.

Four different hops extracts (“Hops 1–4”) and three different hemp extracts (“Hemp 1–3”) were evaluated for their impact on cellular viability and anti-inflammatory potency in RAW 264.7 mouse macrophages. The chemical composition of all extracts was provided by the manufacturers (see Additional file 1: Tab. S1–S3). Among the four hops extracts, Hops 1 is particularly high in terpene content [781 mg/g]. The terpene fraction in Hops 1 is dominated by three terpenes: β-myrcene [350 mg/g], α-humulene [315 mg/g], and β-caryophyllene [85 mg/g] (see Additional file 1: Tab. S1).

Although these terpenes are also present in Hemp 1, they are part of a broader array of terpenes, including α-/β-pinene, limonene, and α-bisabolol (see Additional file 1: Tab. S2). Additionally, the three hemp extracts exhibited notable differences in their cannabinoid profiles, with Hemp 3 showing a much lower CBD content [3.44% (w/w)] than Hemp 1 and Hemp 2 [92.96% (w/w) and 90.86% (w/w), respectively].

### In vitro cytotoxicity test

**Fig. 1:**
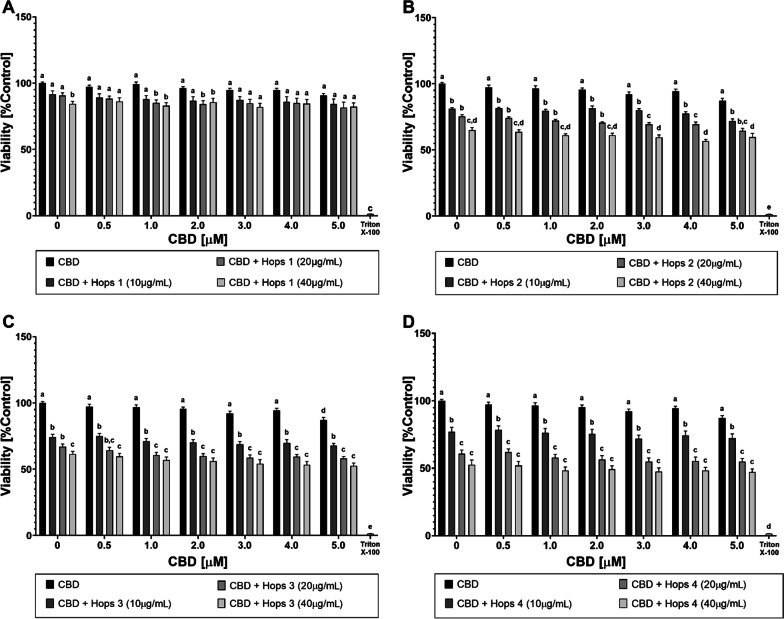
Influence of synthetic CBD in combination with different hops extracts on cell viability of LPS-stimulated RAW 264.7 cells. Relative cell viability of RAW 264.7 mouse macrophages after incubation with synthetic CBD in combination with each of the Hops 1–4 extracts (**a–d**). Control: DMEM (− PR, + 1% P/S, + 10% FBS), 1% (v/v) DMSO, 100 ng/mL LPS. Incubation with Triton X-100 [0.1% (v/v)] results in < 5% cell viability. All graphs show means ± SEM from at least 3 independent experiments. Significantly different means within one graph do not share same letters (two-way ANOVA with Tukey’s multiple comparisons post-hoc test, p < 0.05)

Initially, the cell viability-modulating effects of the individual components CBD, Hops 1 extract, and hydrocortisone in the presence or absence of LPS stimulation were investigated by metabolic activity measurements (see Additional file 1: Fig. S1). The cytotoxicity of CBD was calculated to be IC_50_CBD = 15.44 ± 0.44 µM. For hops 1 and hydrocortisone, significantly weaker viability-reducing effects were observed in this prescreening and the 50% threshold was not reached. Subsequently, cell viability analyses were carried out to evaluate the combined effects of CBD [0–5 µM] and Hops 1–4 extracts [0–40 µg/mL] added prior to stimulation with LPS [100 ng/mL] (Fig. 1a–d). CBD treatment alone with subsequent LPS stimulation did not significantly impact the cellular viability of RAW 264.7 in three out of four experimental setups (Fig. 1a, b, d). However, pretreatment with any of the hops 2–4 extracts in the absence of CBD caused dose-dependent cytotoxicity of up to 50% compared to cells stimulated with LPS alone (Fig. 1b–d). Introducing CBD together with Hops 1 prior to LPS treatment resulted in much lower cytotoxicity, with RAW 264.7 cellular viability of > 80% even at the highest concentrations of 5 µM CBD and 40 µg/mL Hops 1 extract (Fig. 1a).

**Fig. 2:**
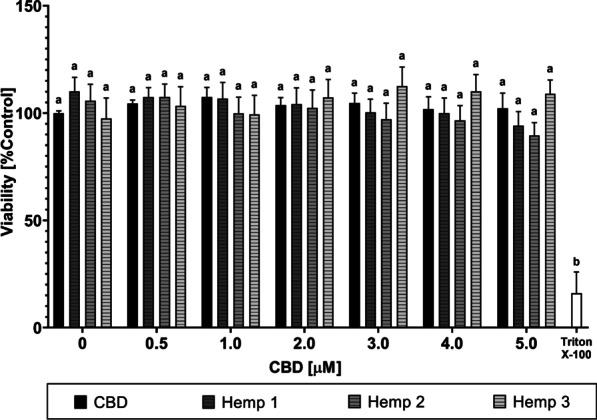
Influence of synthetic CBD versus hemp extracts on cell viability of LPS-stimulated RAW 264.7 cells. Relative cell viability of RAW 264.7 mouse macrophages after incubation with synthetic CBD or Hemp 1–3 extracts of equimolar CBD content. The CBD concentration on the x-axis represents the final CBD content in the experimental setup, either retrieved from the synthetic CBD or the different hemp extracts. Control: DMEM (− PR, + 1% P/S, + 10% FBS), 1% (v/v) DMSO, 100 ng/mL LPS. Incubation with Triton X-100 [0.1% (v/v)] served as a positive control for maximum cytotoxicity. All data show means ± SEM from at least three independent experiments. Significantly different means within one graph do not share same letters (two-way ANOVA with Tukey’s multiple comparisons post-hoc test, p < 0.05)

In order to explore the potential of synthetic CBD-hops extract combinations as a viable alternative to cannabis-based medications for anti-inflammatory purposes, hemp extracts were evaluated in parallel. Hemp extracts are a natural source of terpenes and non-psychotropic plant cannabinoids, particularly CBD (see Additional file 1: Tab. S2 and S3 and [44]). Therefore, three different hemp extracts (Hemp 1–3) were evaluated with regard to their cytotoxicity on RAW 264.7 cells by applying either pure CBD or the three hemp extracts at equimolar CBD concentrations [0–5 µM]. As depicted in Fig. 2, no significant differences in cellular viability were observed after treatment with CBD and LPS in the presence of hemp extracts 1–3 compared to cells stimulated with LPS alone.

### Effect of synthetic CBD in combination with hops extract compared to hemp extract on the NO· release by LPS-stimulated RAW 264.7 macrophages

To determine the anti-inflammatory potential of CBD and hops extract, their effect on the release of NO· in LPS-stimulated RAW 264.7 mouse macrophages was investigated using the Griess assay. Upon stimulation with LPS, nitrite, the stable oxidation product of NO·, was detected at concentrations of roughly 55 µM in cell supernatants (see Additional file 1: Fig. S2), consistent with previous studies using this mouse macrophage cell model [45, 46]. Pre-incubation with hydrocortisone resulted in a dose-dependent reduction of nitrite, with a maximum reduction to approximately 20% of the LPS-stimulated control at 100 µM hydrocortisone (see Additional file 1: Fig. S3). However, the results of the MTT tests (see Additional file 1: Fig. S1) led to the decision to use hydrocortisone as an anti-inflammatory control at a lower concentration of 50 µM in subsequent experiments.

**Fig. 3:**
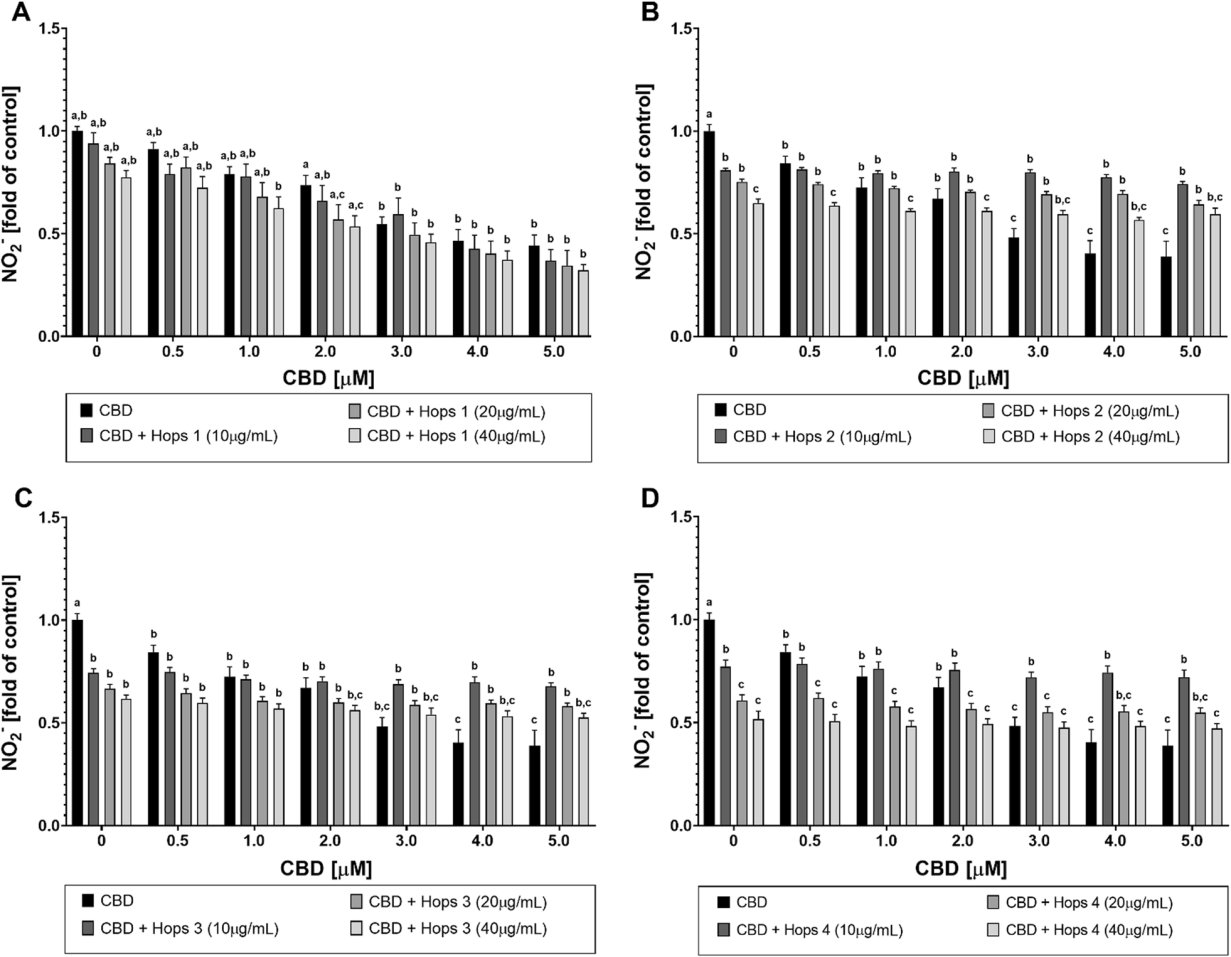
Influence of synthetic CBD in combination with hops extract on LPS-induced nitric oxide production in RAW 264.7 cells. Relative amount of the stable NO· oxidation product nitrite in the supernatants of RAW 264.7 mouse macrophages after incubation with synthetic CBD in combination with Hops 1–4 extracts and subsequent stimulation with LPS. Control: DMEM (− PR, + 1% P/S, + 10% FBS), 1% (v/v) DMSO, 100 ng/mL LPS. All graphs show means ± SEM from a minimum of 3 independent experiments. Significantly different means within one graph do not share same letters (two-way ANOVA with Tukey’s multiple comparisons post-hoc test, p < 0.05)

Figure 3a–d shows the results of nitrite quantification after treatment with CBD and hops extracts (Hops 1–4) and subsequent stimulation with LPS [100 ng/mL]. CBD alone exhibited anti-inflammatory activity in LPS-stimulated RAW 264.7 cells, with a maximum reduction of NO· levels to < 50% of the LPS control, which was comparable to the efficiency of hydrocortisone [50 µM]. Hops 1 demonstrated a certain anti-inflammatory tendency alone and in combination with CBD under TLR4 stimulation, although these effects were not statistically significant (Fig. 3a). Treatment with CBD in combination with Hops 2–4 resulted in fundamentally different outcomes. Again, in this experimental setup, CBD dose-dependently decreased LPS-induced nitrite levels. Hops extracts 2–4 alone reduced NO· release and exerted anti-inflammatory effects, as shown by the Griess data. However, the combination of CBD with these extracts under TLR4-stimulated conditions led to higher nitrite values than the application of CBD alone (Fig. 3b–d). In this regard, the results from the cytotoxicity studies should be considered carefully, as the increased cytotoxicity of these incubation variants might explain the higher NO· levels found in cell supernatants (Fig. 1a–d). These findings prompted us to focus on Hops extract 1 in further experiments.

**Fig. 4:**
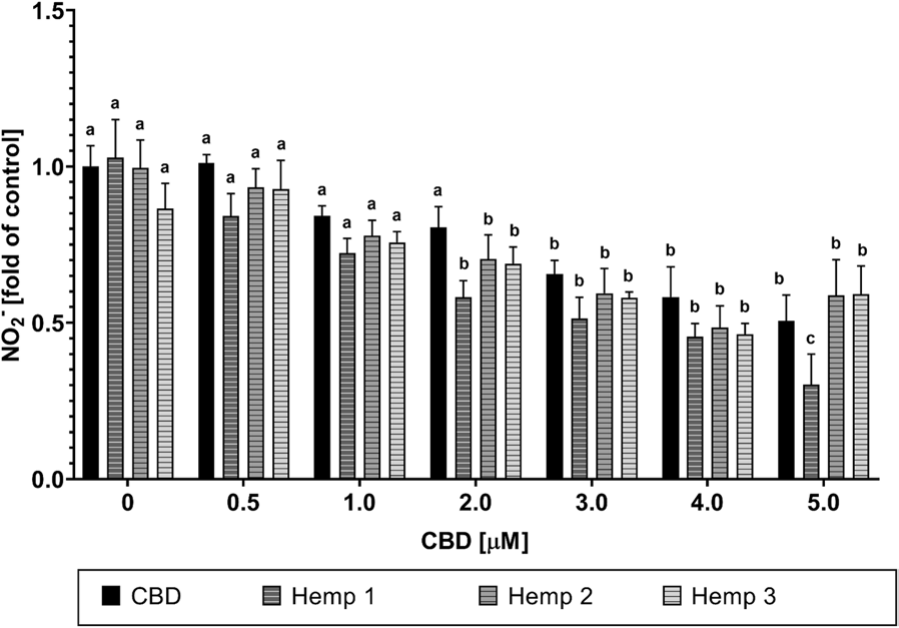
Influence of synthetic CBD versus hemp extracts on LPS-induced nitric oxide production in RAW 264.7 cells. Relative amount of the stable NO· oxidation product nitrite in the supernatants of RAW 264.7 mouse macrophages after incubation with synthetic CBD or different hemp extracts and subsequent stimulation with LPS. Control: DMEM (− PR, + 1% P/S, + 10% FBS), 1% (v/v) DMSO, 100 ng/mL LPS. All data show means ± SEM from a minimum of 3 independent experiments. Significantly different means within one graph do not share same letters (two-way ANOVA with Tukey’s multiple comparisons post-hoc test, p < 0.05)

The Griess assay was also applied to supernatants from LPS-stimulated RAW 264.7 cells after treatment with CBD and Hemp 1–3 at equimolar CBD concentration ranging from 0 to 5 µM and LPS stimulation (see Fig. 4). Again, CBD led to a dose-dependent reduction of extracellular nitrite in LPS-stimulated cells. Treatment with Hemp 1–3 was similar in efficacy with a significant difference for Hemp 1, which showed a more pronounced effect in reducing NO· levels, especially when applied at higher CBD concentrations [5 µM]. Due to these results, Hemp extract 1 was selected for further investigations.

### Effects of CBD in combination with hops extract compared to hemp extract on the production of pro-inflammatory cytokines in RAW 264.7 cells

**Fig. 5:**
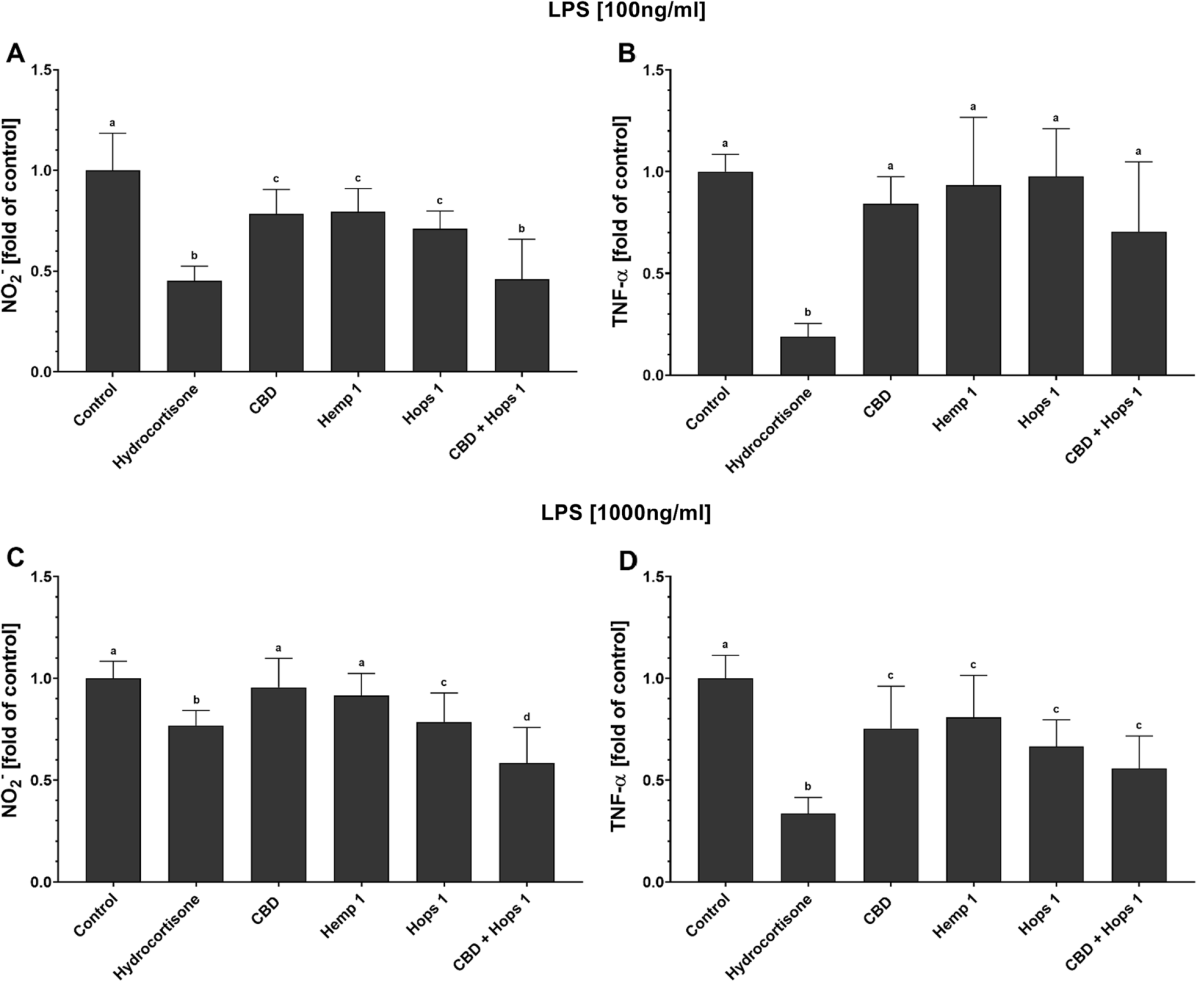
Inflammatory markers produced by RAW 264.7 cells after stimulation with LPS. RAW 264.7 mouse macrophages were treated with the test substances prior to TLR4 stimulation with (**a, b**) 100 ng/mL or (**c, d**) 1000 ng/mL LPS. Anti-inflammatory effects were assessed as changes in (**a, c**) nitrite or (**b, d**) TNF-α in cell culture supernatant relative to the respective untreated, LPS-stimulated control. Control: DMEM (− PR, + 1% P/S, + 10% FBS), 1% (v/v) DMSO, LPS [100 ng/mL] (**a, b**) or [1000 ng/mL] (**c, d**). All data show means ± SEM from a minimum of 3 independent experiments. Significantly different means within one graph do not share same letters (one-way ANOVA with Tukey’s multiple comparisons post-hoc test, p < 0.05)

In addition to NO·, RAW 264.7 cells also release several pro-inflammatory cytokines in response to TLR4 stimulation with LPS [47]. In this study, TNF-α was assessed as a second inflammatory marker (Fig. 5). Stimulation with 100 ng/mL LPS resulted in average TNF-α levels of approximately 260 ng/mL, almost tenfold higher than the levels observed in the absence of LPS. Increasing the LPS concentration to 1000 ng/mL led to a minimal further increase in TNF-α to around 300 ng/mL (see Additional file 1: Fig. S2).

Following stimulation with 100 ng/mL LPS, TNF-α measurements showed a similar trend compared to the release of NO·. Furthermore, at 1000 ng/mL LPS-stimulation the combination of CBD [5 µM] and Hops 1 [40 µg/mL] also showed the strongest anti-inflammatory effects with respect to NO·, approximately 60% relative to the LPS-stimulated RAW 264.7 controls. In comparison, hydrocortisone [50 µM] exhibited weaker anti-inflammatory effects of under this treatment regime, resulting in approximately 25% reduction in nitrite. Again, this anti-inflammatory effect was also evident in a less pronounced gradation in the TNF-α measurements. At both LPS concentrations, the positive control, hydrocortisone, shows a strong anti-inflammatory effect, which is particularly pronounced for the reduction in TNF-α levels.

### CBD uptake capability of RAW 264.7 mouse macrophages

Tab. 1 presents the CBD levels found in the cell extracts of LPS-stimulated RAW 264.7 mouse macrophages after incubation with CBD, Hemp 1, or a combination of CBD and Hops 1. Although all three experimental groups were treated with the same CBD concentrations [5 µM], the cellular CBD levels varied with increasing terpene concentrations. Specifically, the CBD levels of LPS-stimulated RAW 264.7 after application of Hemp 1 were increased by about 29% compared to the concentrations of the CBD-treatment group. Furthermore, the combination of CBD and Hops extract 1 resulted in an increase of about 79%, a significant increase compared to the application of equimolar levels of pure CBD (Table 1).

**Table 1:** CBD levels in cell extracts from LPS-stimulated RAW 264.7 cells after treatment with CBD and different plant extracts

Test substance	Mean ± SEM [ng/mg protein]
CBD [5 µM]	79.54 ± 10.42^a^
Hemp 1 [5 µM]*	102.8 ± 6.81^a,b^
CBD [5 µM] + Hops 1 [40 µg/mL]	142.9 ± 15.34^b^

Data are shown as means ± SEM of a minimum of 4 different experiments. Significantly different means within one graph do not share same letters (one-way ANOVA with Tukey’s multiple comparisons post-hoc test, p < 0.05)

*Equimolar CBD concentration [5 µM]

## Discussion

This study aimed to explore the potential application of a combined formulation of cannabidiol and secondary metabolites from either *C. sativa* or *H. lupulus* extracts for long-term treatment of inflammatory diseases. CBD alone has already shown promising results in the treatment of several inflammatory diseases, including inflammatory skin conditions [6, 48–50], inflammatory bowel disease [51], type 1 diabetes [52], and osteoarthritis [53]. However, its therapeutic potential still requires further investigations to improve cellular availability and to further identify other phytochemicals that contribute to the previously mentioned “entourage effect”, which potentially strengthen its effect on cellular targets [54, 55]. The proposed entourage effect encompasses the effects of both cannabinoids and terpenes [28, 56], which led to the idea of combining pure CBD with a source of terpenes that is related to the cannabis plant, but does not produce cannabinoids. Therefore, hops was chosen as a suitable alternative source of terpenes (see Additional file 1: Table S1). Additionally, a broad-range hemp extract was used to compare the effects of the CBD-hops combination to an extract from *C. sativa*, containing a wide range of terpenes besides the cannabinoid content (see Additional file 1: Table S2 and S3). The cytotoxicity after pre-incubation with the different test substances and extracts, followed by stimulation with LPS, was measured in MTT assays (see Figs. 1 and 2) and the levels of NO· and TNF-α in cell supernatants were measured by Griess assays (see Figs. 3 and 4) and sandwich ELISAs (see Fig. 5), respectively. Finally, the cellular CBD uptake in the presence of terpenes was determined by LC–MS (see Table 1).

Numerous publications have demonstrated the potent anti-inflammatory effect of CBD both in-vitro and in-vivo [13, 57, 58]. The underlying mechanisms involve direct suppression of the activation of different types of immune cells, induction of apoptosis, and promotion of regulatory cells, which in turn control other immune cell targets. CBD’s mode of action includes activation of cannabinoid and other receptors, inhibition of cytokines and cell proliferation, as well as induction of apoptosis [59, 60].

Inflammation occurs when innate immune cells recognize pathogens, injury, or danger signals via pattern recognition receptors and activate intracellular signaling cascades to produce mediators locally controlling the immune response. TLR4 is a pattern recognition receptor acting as a lipopolysaccharide (LPS) sensor. Its activation results in the production of several pro-inflammatory, antiviral, and anti-bacterial cytokines [61]. Additionally, RAW 264.7 cells also express CB1/CB2 receptors [62], making this cell line a suitable model to study the interaction between the cannabinoid system and Toll-like receptor control of inflammation [63].

In the applied concentrations, CBD [0–5 µM] exerted minimal cytotoxic effects in LPS-stimulated RAW 264.7 mouse macrophages. These findings confirm the results of several other studies using isolated CBD in this murine cell line [43, 64], and therefore the highest concentration [5 µM] was chosen for further experiments. Similar to the present study, Silva et al. determined the IC_50_ for CBD in RAW 264.7 at a concentration of 15 µM [64]. Plasma CBD levels in dogs and rodents are reported between 0.01 and 5 μM [54, 65]. Additionally, the bioavailability and metabolites of cannabidiol in humans have recently been reviewed, and the highest systemic CBD levels were reported following i.v. administration of 20 mg deuterium-labeled CBD at ~ 2.2 µM plasma CBD (t_max_ = 3 min), which dropped to 0.2 µM after 1 h [66].

The present study shows a dose-dependent anti-inflammatory potency of CBD, as evidenced by the reduction in NO· and TNF-α release from LPS-stimulated RAW 264.7 cells. Similar findings have been reported in other cellular experimental setups using different cell types. For instance, Danchine (2020) demonstrated the anti-inflammatory effect of CBD [0.2 µM or 20 µM] on LPS-stimulated RAW 264.7 mouse macrophages. Although the authors did not observe a significant effect of CBD on NO· levels, the quantification of IL-6 indicated a strong anti-inflammatory effect of CBD [20 µM], decreasing levels of the cytokine in cell supernatants by approximately 75% [42]. Recent studies have also evaluated the anti-inflammatory efficacy of CBD and its variants as a multifunctional cosmetic raw material [67]. Moreover, Muthumalage and Rahman (2019) studied the LPS-induced inflammatory response of different monocyte cell lines and found a substantial decrease in cytokine release and NF-κB activity upon treatment with CBD [10.6–42.4 µM] [43]. Furthermore, in an experimental model of allergic contact dermatitis on [poly-(I:C)]–stimulated human keratinocytes (HaCaT), CBD [5–20 µM] showed a dose-dependent anti-inflammatory effect on several biochemical markers, including MCP-2, IL-6, IL-8, and TNF-α, while simultaneously increasing cellular production of the endocannabinoid anandamide (AEA). This effect could be reversed by the addition of CB_2_ (AM630) and TRPV1 (I-RTX) receptor antagonists [68], indicating the involvement of these receptors in the anti-inflammatory signaling cascade triggered by CBD in keratinocytes. CBD is classified as a multi-target modulator because it acts on several cellular structures, including agonistic effects on PPARγ (peroxisome proliferator-activated receptor gamma), 5-HT_1A_ (serotonin 1A receptor), and several GABA (gamma-aminobutyric acid) receptors, as well as antagonistic effects on nAChRs (nicotinic acetylcholine receptors), TRPM8 (transient receptor potential cation channel subfamily M), and FAAH-1, and -2 (fatty acid amid hydrolase 1 and 2), which in turn results in the upregulation of the endocannabinoids AEA (anandamide) and 2-AG (2-arachidonylglycerol) [57, 69].

Albeit the entourage effect is still controversial, it may offer a valuable insight into the future of cannabis-based therapy [26–29, 70]. In an effort to enhance the cellular effects of CBD, we decided to add terpene-rich extracts from *H. lupulus* to the CBD treatment. The anti-inflammatory potential of several well-known terpenes, including limonene [35, 71], β-caryophyllene [72, 73], and β-myrcene [74, 75], has been established in recent years. In a 2018 report by Gallily et al., the anti-inflammatory potential of terpenoid extracts derived from *C. sativa* [5–40 µg/mL] was demonstrated by a reduction in NO· levels following application onto LPS-stimulated RAW 264.7 cells. However, the same study reported no significant effect on TNF-α levels in an in vivo model of zymosan-induced paw swelling in mice [76]. In this study, we opted to use terpenes derived from *H. lupulus* (hops) as an alternative source, in order to avoid the possibility of THC residues in the extracts.

The chemical analysis of all hops extracts shows a clear distinction between Hops 1 and Hops 2–4 in their respective terpene concentration (see Additional file 1: Tab. S1). Compared to Hops 1, the terpene fraction in Hops 2–4 is much lower, resulting in a higher fraction of other phytochemicals, such as α- and β-bitter acids. These bitter acids have already been detected in ethanolic extracts of various *H. lupus* cultivars in previous studies and were related to proliferation inhibition in prostate (PC3) and colon (HT29) cancer cell lines at concentrations comparable to those of hops extracts Hops 2–4 [mean α- and β-bitter acids in Hops 2–4: 24.47% (w/w)], unlike the lower concentrations found in Hops 1 [mean α– and β-bitter acids in Hops 1: 4.7% (w/w)] [77]. Due to its low cellular toxicity and superior effectiveness in restricting NO· formation, Hops extract 1 was chosen in this study for further experiments.

The gas chromatographic analysis commissioned for Hops 1 revealed the presence of three predominant terpenes in the extract: β-myrcene [350 mg/g], α-humulene [315 mg/g], and β-caryophyllene [85 mg/g], which constituted more than 96% of the total terpene fraction (see Additional file 1: Table S1). Amongst these, β-caryophyllene has received considerable attention in recent years due to its CB_2_ agonistic effects, which are not usually attributed to terpenes [78]. β-caryophyllene has also been found to interact with PPAR ligand-activated nuclear receptors, leading to the direct activation of PPARα [79] and CB_2_-mediated activation of PPARγ [80], thereby contributing to its substantial anti-inflammatory activity. β-myrcene has also exhibited substantial anti-inflammatory and anti-oxidative activity in several in vitro studies [74, 81], as well as an animal model of diabetes mellitus, where it was found to inhibit NF-κΒ-mediated inflammatory cytokines and pro-inflammatory signaling [75]. Unlike β-caryophyllene, β-myrcene does not bind to cannabinoid receptors, but exerts its bioactivity through other mechanisms, which remain incompletely understood to date [82]. Lastly, the monocyclic sesquiterpene α-humulene has demonstrated promising anti-inflammatory potential and, in some cases, may even be superior to β-caryophyllene, as demonstrated in various inflammatory models in mice and rats by Fernandes et al. [83]. In that same study, both β-caryophyllene ((−)-trans-caryophyllene) and α-humulene achieved comparable protective effects to dexamethasone in the control group.

Furthermore, a recent study by LaVigne et al. suggests a partial activity of α-humulene on the CB_1_ receptor, resulting in a selective enhancement of cannabinoid activity, supporting the recent discussion of potential synergies between terpenes and cannabinoids found in the *C. sativa* plant [41].

Several research groups have investigated the combination of CBD with either pure phytomolecules or complex plant extracts. For instance, Rajan et al. reported on the anti-inflammatory effects of a combination of CBD [5 µM] and moringin [5 µM] (derived from *Moringa oleifera* seeds) in LPS-stimulated RAW 264.7. The combined application of CBD and moringin demonstrated superior anti-inflammatory and anti-oxidative effects compared to the single constituents [26]. Similar findings were observed in the present study, where the combination of CBD and Hops 1 extract exhibited higher anti-inflammatory efficacy toward LPS-stimulated RAW 264.7 than the single constituents (see Fig. 5). In an extensive review on chemical synergies found in *C. sativa*, Lewis et al. proposed that the terpene profile of a unique hemp chemovar may enable fine-tuning of the pharmacological effects of cannabinoids in a unique manner [84]. Among the hops extracts used in the present study, Hops 1 was notable for its terpene richness, significantly better cell tolerance, and anti-inflammatory properties. The combination of CBD and Hops extract 1 was not only superior to the administration of CBD alone in this experimental set-up but also advantageous over the use of broad-spectrum hemp extracts, which may contain THC residues and thus pose a legal threat to manufacturers and resellers. This risk does not apply when synthetic CBD is combined with isolated phytochemicals or terpene-rich extracts from plants that do not produce cannabinoids, such as *H. lupulus*.

**Fig. 6:**
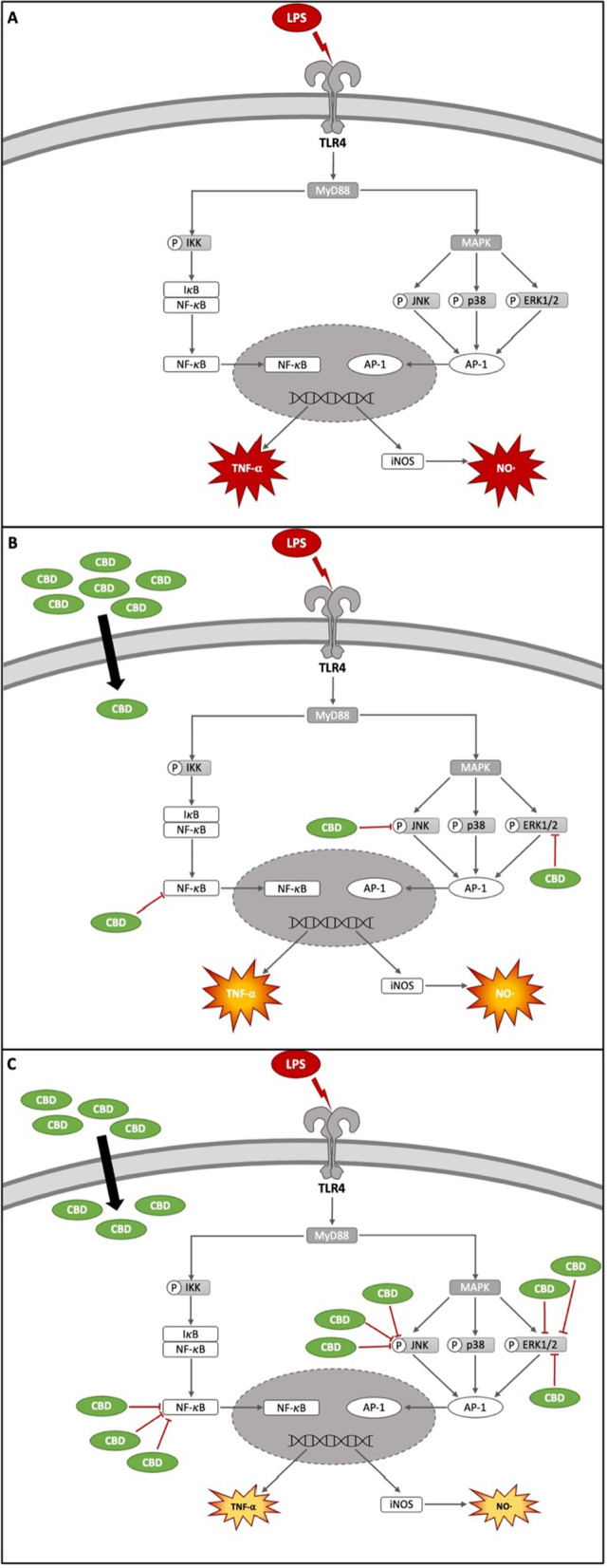
LPS activates inflammatory TLR 4 signaling cascades, in particular those involving NFκB and mitogen-activated protein kinases (MAPK), leading to the production of TNF-α and NO· [59, 95] (**a**). CBD inhibits the inflammatory signaling cascades at several points and decreases the amount of TNF-α and NO· produced in response to LPS stimulation [16] (**b**). The conducted experiments showed that the addition of terpenes from Hops 1 extract increases cellular availability of CBD and thus supports its anti-inflammatory effect (**c**)

Terpenes have been shown to interact with various cellular receptors associated with CBD bioactivity, and they may also aid the cellular availability of CBD. CBD formulations that include terpene blends have the potential to increase cellular CBD absorption, e.g., by affecting the intercellular lipid arrangement in the skin's most outer layer, the stratum corneum [85, 86]. Furthermore, it is necessary to investigate to what extent they alter the expression of ABC transporters and voltage-dependent anion channels, which are believed to be involved in macrophage CBD membrane trafficking [9]. In a previous study, primary keratinocytes had approximately 20 µg CBD per mg of protein in the cytosolic fraction after 24 h of treatment with 4 µM CBD [87]. However, the RAW 264.7 mouse macrophage levels after similar CBD exposure in this study are much lower; still, the addition of Hemp 1 and even more pronounced Hops 1 extract increased cellular CBD availability. The enhanced anti-inflammatory effect observed after combined CBD and hops extract 1 treatment may be partly due to CBD being enhanced for (intra-)cellular targets under these incubation conditions (Fig. 6a–c). Future investigations will help to understand, which of the terpenes contained in Hops extract 1 are essential in this regard by themselves or in interaction with other extract components. Previous investigations of the entourage effect have primarily focused on the possible synergistic modulation of cellular targets by the different compounds of the cannabis plant, i.e., cannabinoids and terpenes, which could then lead to overall enhanced effectiveness [40]. This study, however, adds another aspect to the discussion by demonstrating higher cellular availability of CBD when combined with phytomolecules (Fig. 6c). This could also explain why several terpenes predominant in *C. sativa*, despite not showing activity at the same receptors as cannabinoids, develop their efficacy in different areas of application [27, 82].

In summary, this study supports the evidence of CBD against LPS-induced inflammation, which can be used as an alternative treatment for various inflammatory conditions to commonly used steroid-based treatments that often cause severe side effects. The anti-inflammatory effect of CBD can be strengthened by incorporating terpenoid assemblages or isolated terpenes from non-cannabinoid sources, such as hops, in order to avoid possible residues of psychotropic and strictly regulated THC. The results of this study contribute another aspect to the hypothesized mode of action by which terpenoid assemblages may increase CBDs anti-inflammatory potential. The commonly proposed theory is that the entourage effect is based on a synergistic activity of both terpenes and cannabinoids on the same receptors, whereas we claim a substantial increase in CBDs cellular bioavailability as an additional and important factor for the increased anti-inflammatory activity. This study's results have the potential to shape future therapeutic approaches for alleviating the burden of people with acute or chronic inflammatory conditions.

## Materials and methods

### Reagents

DMSO (≥ 99.9%) was purchased from Sigma-Aldrich (Steinheim, Germany) and used as a solvent for all plant extracts and test substances. Synthetic CBD [99.99%] (PureForm CBD/AP-5478) was obtained from PureForm Global Inc. and dissolved in DMSO as a 10 mM stock solution. Hydrocortisone [≥ 98%] was purchased from Sigma-Aldrich (Steinheim, Germany) and dissolved in DMSO [10 mM].

Hops CO_2_ supercritical extract type 081.001 (Hops 1: charge nr. 602406; Hops 2: charge nr. 491105; Hops 3: charge nr. 371206; Hops 4: charge nr. 881221) were provided by Flavex Naturextrakte GmbH (Rehlingen-Siersburg, Germany) and dissolved in DMSO as 100 mg/mL stocks. Hemp extracts (Hemp 1: charge nr. HS.TL031.BA.30; Hemp 2: charge nr. HS271.BA.10; Hemp 3: charge nr. BA-EKO_0119) were produced by BAFA Neu GmbH (Malsch, Germany) and dissolved in DMSO as 10 mM CBD stocks.

For the identification and quantification of the predominant terpenes in hemp and hops, accredited gas chromatographic methods according to the AOAC guideline [88] were used by the manufacturers.

### Cell culture

The RAW 264.7 mouse macrophage cell line was originally isolated from the ascites fluid of a male mouse that was infected intraperitoneally with Abelson-murine leukemia virus [89]. Cells were grown in Dulbecco’s Modified Eagle Medium (DMEM) containing phenol red (+ PR) as a pH indicator, supplemented with 10% fetal bovine serum (FBS) and 1% P/S (Penicillin [100 U/mL]/Streptomycin [100 μg/mL]) in TC-75 cell culture flasks. Cells were incubated at 37 °C in 5% CO_2_ atmosphere and passaged every 2–3 days.

### MTT assay

For determination of cell viability, 50,000 cells/well were seeded in 96-well plates and incubated overnight at 37 °C and 5% CO_2_. On the next day, the medium was exchanged to 100 μL/well DMEM (without phenol red (− PR), + 1% P/S, + 10% FBS) plus 50 μL/well of the individual test substances or extracts from fourfold stocks [CBD 0–20 µM], Hops 1 [0–160 µg/mL], CBD [0–20 µM] + Hops 1 [0–160 µg/mL], Hemp 1 [0–20 µM], Hydrocortisone [0–400 µM], Triton X-100 [0.4% (v/v)]. Following 4 h of pre-incubation at 37 °C and 5% CO_2_, 50 μL/well of 400 ng/mL LPS (final concentration per cavity: 100 ng/mL) or medium as control was added and the cells were incubated for another 20 h. After 24 h, 50 μL of the supernatants from each well were transferred to a new 96-well-plate and later used for the analysis of nitrite levels as a marker for nitric oxide (NO·) production in a Griess assay (see 4.4 Griess Assay). The remaining supernatant was discarded, and cell layers were used for metabolic activity measurements by applying 0.5 mg/mL MTT solution in DMEM (− PR, + 1% P/S, + 10% FBS) [90]. The plate was then incubated at 37 °C and 5% CO2 for 1 h before MTT removal and cell lysis (100 μL/well lysis reagent (EtOH/DMSO [50% (v/v)]). After an additional 30 min of shaking in the dark, the purple color indicating cell viability was quantified with a spectrometer (TECAN^®^ infinite M 200) at 570 nm (reference: 630 nm), and cell viability was calculated as percent of the untreated controls (DMEM (− PR, + 1% P/S, + 10% FBS) + 1% DMSO + 100 ng/mL LPS) [90].

### Griess assay

The Griess assay [91] was performed to quantify nitrite levels in supernatants of RAW 264.7 cells treated with test substances and extracts and stimulation with LPS, as previously reported by Keil et al. for the screening of anti-inflammatory agents [92]. The cell supernatants collected before measuring cellular metabolic activity (see 4.4 MTT Assay) were mixed with 50 μL of sulfanilamide solution [1% (w/v) in 5% H_3_PO_4_] and the plate was incubated in the dark. After 10 min, 50 μL of NED solution [0.1% (w/v) in H_2_O bidest.] was added to each cavity and the plate was kept in the dark for another 10 min. The resulting color intensity was measured spectrophotometrically (TECAN^®^ infinite M 200) at 520 nm (reference: 612 nm) and calculated as absolute NaNO_2_ concentrations using an external calibration curve [0–50 µM NaNO_2_]. The results are expressed as fold increase in NaNO2 levels compared to the solely LPS-stimulated cells (DMEM (− PR, + 1% P/S, + 10% FBS) + 1% DMSO + 100 ng/mL LPS).

### ELISA assay

A sandwich-type ELISA was used to quantify TNF-α as a proinflammatory cytokine in lipopolysaccharide-stimulated RAW 264.7 mouse macrophages. 2.75 × 10^6^ cells/well were seeded in 24-well plates and incubated at 37 °C and 5% CO_2_ overnight. The medium was replaced by 250 μL/well of DMEM (− PR, + 1% P/S, + 10% FBS) plus 125 μL of fourfold concentrated test substances or extracts. After 4 h of pre-incubation at 37 °C and 5% CO_2_, 125 μL of 400 ng/mL or 4000 ng/mL LPS (final concentrations: 100 ng/mL and 1000 ng/mL, respectively) was added, and the plate was incubated at 37 °C and 5% CO_2_ for another 20 h. LPS concentrations were chosen according to studies by Takahashi et al. (2012) and Yoon et al. (2010). The cell supernatants were collected after 48 h and analyzed in a Griess assay to substantiate inflammatory response before analyzing the supernatants for TNF-α content in a sandwich ELISA. The experimental procedure was followed according to the instructions given by the manufacturer of the Mouse TNF (mono/mono) ELISA Set (Cat. 555268; Lot.: 9078995, BD Biosciences, San Diego, California) [93].

### Cellular uptake of CBD

For the analysis of RAW 264.7 CBD uptake capability, 3 × 10^6^ cells were seeded in 10 mL medium [DMEM (+ PR, + 1% P/S, + 10% FBS)] into 10 cm round cell culture dishes on day 1 of each experiment. The cells were incubated at 37 °C and 5% CO_2_ for 24 h to allow adherence. The medium was then removed and exchanged with fresh medium (DMEM (− PR, + 1% P/S, + 10% FBS)) with CBD [5 µM], Hemp 1 [c(CBD) = 5 µM], or a combination of CBD [5 µM] and Hops 1 [40 µg/mL]. The final volume in each cell culture dish was 15 mL. After 24 h of incubation, the medium containing test substances was removed from each plate and the cells were trypsinized and resuspended in 10 mL of fresh medium [DMEM (− PR, + 1% P/S, + 10% FBS)] each. The cells were centrifuged for 5 min at 350 g, and the supernatants were removed carefully. The cell pellets were resuspended in PBS (1 mL) and centrifuged again (4 °C, 5 min, 400 g). Finally, the supernatants were removed again, and the cell pellets were stored at − 80 °C until further analysis.

Cellular CBD was quantified using ultra-performing liquid chromatography-tandem mass spectrometry (LCMS 8060, Shimadzu, Kyoto, Japan) [94]. Cell lysates obtained by sonification were centrifuged (4 °C, 10 min, 15,000 g) and the supernatant containing the cytosolic fraction was separated from the membrane fraction. CBD was extracted from samples using SPE, separated on Poroshell 120 EC-C18 column (3.0 mm × 150 mm, 2.7-micron) as described above, and analyzed in the positive-ion mode (MRM). CBD-d_9_ was used as an internal standard for quantification. The precursor to the product ion transition was 315.1 → 193.00 for CBD. The level of CBD was expressed in micrograms per milligram of protein.

### Statistical analysis

All statistical analyses were conducted using GraphPad Prism version 8.0 (GraphPad Software, San Diego, CA, USA). Data were plotted as means ± SEM of a minimum of three independent experiments.

## Supplementary Information

**Additional file 1: Table S1.** Terpene Profiles of Hops 1–4. **Table S2.** Terpene Profiles of Hemp 1–3. **Table S3.** Cannabinoid Profiles of Hemp 1–3. **Fig. S1.** Toxicity assessment of CBD, hydrocortisone, and Hops 1 extract in cells RAW 264.7 cells. **Fig. S2.** Amount of nitrite and TNF-α in RAW 264.7 supernatants after LPS stimulation. **Fig. S3.** Influence of hydrocortisone/LPS-treatment on nitric oxide production in RAW 264.7 cells.

## Data Availability

The datasets generated during and/or analyzed during the current study are available from the corresponding author on reasonable request.
