# Supplementary material for: Effects of combined cannabidiol (CBD) and hops (Humulus lupulus) terpene extract treatment on RAW 264.7 macrophage viability and inflammatory markers
Source: Nat Prod Bioprospect. 2023 Jun 7;13(1):19. doi: 10.1007/s13659-023-00382-3 (PMC10247647; doi:10.1007/s13659-023-00382-3)
Supplement: Supplementary file 1 — Additional file 1: Table S1. Terpene Profiles of Hops 1–4. Table S2. Terpene Profiles of Hemp 1–3. Table S3. Cannabinoid Profiles of Hemp 1–3. Fig. S1. Toxicity assessment of CBD, hydrocortisone, and Hops 1 extract in cells RAW 264.7 cells. Fig. S2. Amount of nitrite and TNF-α in RAW 264.7 supernatants after LPS stimulation. Fig. S3. Influence of hydrocortisone/LPS-treatment on nitric oxide production in RAW 264.7 cells. [file 13659_2023_382_MOESM1_ESM.docx]

Effects of Combined Cannabidiol (CBD) and Hops (*H. lupulus*) Terpene Extract Treatment on RAW 264.7 Macrophage Viability and Inflammatory Markers

Inga Dammann^a*†^, Claudia Keil^b*^, Iris Hardewig^a^, Elżbieta Skrzydlewska^c^, Michał Biernacki^c^, Hajo Haase^b†^

^a^ Sanity Group GmbH, Jägerstraße 28-31, 10117 Berlin, Germany: [inga.dammann@sanitygroup.com](mailto:inga.dammann@sanitygroup.com); i.hardewig@life-mi.com

^b^ Department of Food Chemistry and Toxicology, Technische Universität Berlin, Straße des 17. Juni 135, 10623 Berlin, Germany: [c.keil@tu-berlin.de](mailto:c.keil@tu-berlin.de); haase@tu-berlin.de

^c^ [Department of Analytical Chemistry, Medical University of Bialystok, A. Mickiewicza 2D, 15-222 Bialystok, Poland: elzbieta.skrzydlewska@umb.edu.pl](mailto:Department%20of%20Analytical%20Chemistry,%20Medical%20University%20of%20Bialystok,%20A.%20Mickiewicza%202D,%2015-222%20Bialystok,%20Poland:%20elzbieta.skrzydlewska@umb.edu.pl); [michal.biernacki@umb.edu.pl](mailto:michal.biernacki@umb.edu.pl)

***** Contributed equally

† Corresponding authors

**Tab. S1. Terpene Profiles of Hops 1-4.**

| **Terpenes [mg/g]**  **Charge number** | **Hops 1**  **(602406)** | **Hops 2**  **(491105)** | **Hops 3**  **(371206)** | **Hops 4**  **(881221)** |
| --- | --- | --- | --- | --- |
| alpha-Pinene | 1.50 | 0.08 | n.d. | 0.11 |
| Camphene | 0.16 | n.d. | n.d. | n.d. |
| beta-Pinene | 4.40 | 0.63 | 0.61 | 0.73 |
| beta Myrcene | 350.00 | 23.52 | 24.95 | 25.51 |
| ∆3-Carene | 0.03 | n.d. | n.d. | n.d. |
| alpha-Terpinene | n.d. | n.d. | n.d. | n.d. |
| p-Cymene | 0.15 | n.d. | n.d. | n.d. |
| Limonene | 2.20 | n.d. | 0.11 | 0.15 |
| Eucalyptol (1,8-Cineol) | n.d. | n.d. | n.d. | n.d. |
| Ocimene | 1.30 | n.d. | n.d. | n.d. |
| gamma-Terpinene | 0.03 | n.d. | n.d. | n.d. |
| Terpinolene | n.d. | n.d. | n.d. | n.d. |
| Linalool | 3.90 | 0.52 | 0.40 | 0.26 |
| Isopulegol | n.d. | n.d. | n.d. | n.d. |
| Geraniol | 6.40 | n.d. | n.d. | n.d. |
| beta-Caryophyllene | 85.00 | 8.58 | 11.92 | 11.67 |
| alpha-Humulene | 315.00 | 27.87 | 42.19 | 40.33 |
| Nerolidol | 0.32 | n.d. | n.d. | n.d. |
| Caryophyllene oxide | 11.0 | n.d. | 0.18 | 0.13 |
| Guaiol | n.d. | n.d. | n.d. | n.d. |
| alpha-Bisabolol | n.d. | n.d. | n.d. | n.d. |
| **Sum (Terpenes)** | **781** | **61** | **80** | **79** |

n.d. = not detected

**Tab. S2. Terpene Profiles of Hemp 1-3.**

| **Terpenes [mg/g]**  **Charge number** | **Hemp 1**  **(HS.TL031.BA.30)** | **Hemp 2**  **(HS271.BA.10)** | **Hemp 3**  **(BA-EKO_0119)** |
| --- | --- | --- | --- |
| alpha-Pinene | 0.14 | 0.15 | 0.05 |
| Camphene | n.d. | n.d. | n.d. |
| beta-Pinene | 0.13 | 0.15 | 0.02 |
| beta Myrcene | 0.34 | 0.66 | 0.04 |
| ∆3-Carene | n.d. | n.d. | n.d. |
| alpha-Terpinene | n.d. | n.d. | n.d. |
| p-Cymene | 0.04 | 0.09 | 0.07 |
| Limonene | 0.26 | 0.44 | 0.04 |
| Eucalyptol (1,8-Cineol) | n.d. | n.d. | 0.02 |
| Ocimene | n.d. | 0.26 | 0.04 |
| gamma-Terpinene | n.d. | n.d. | 0.01 |
| Terpinolene | n.d. | n.d. | 0.01 |
| Linalool | 0.01 | 0.01 | n.d. |
| Isopulegol | n.d. | n.d. | n.d. |
| Geraniol | n.d. | n.d. | n.d. |
| beta-Caryophyllene | 0.20 | 0.28 | 4.30 |
| alpha-Humulene | 0.07 | 0.08 | 1.20 |
| Nerolidol | 0.09 | 0.10 | 0.10 |
| Caryophyllene oxide | 0.09 | 0.04 | 0.38 |
| Guaiol | 0.10 | 0.10 | n.d. |
| alpha-Bisabolol | 0.18 | 0.14 | 0.19 |
| **Sum (Terpenes)** | **1.60** | **2.51** | **6.46** |

n.d. = not detected

**Tab. S3. Cannabinoid Profiles of Hemp 1-3.**

| **Cannabinoids [%(w/w)]**  **Charge number** | **Hemp 1**  **(HS.TL031.BA.30)** | **Hemp 2**  **(HS271.BA.10)** | **Hemp 3**  **(BA-EKO_0119)** |
| --- | --- | --- | --- |
| ∆9-Tetrahydrocannabinol (THC) | n.d. | n.d. | 0.10 |
| Tetrahydrocannabinolic Acid (THCA) | n.d. | n.d. | n.d. |
| Cannabidiol (CBD) | 93.10 | 90.86 | 3.44 |
| Cannabidiolic Acid (CBDA) | n.d. | n.d. | 0.05 |
| Cannabinol (CBN) | n.d. | n.d. | 0.03 |
| Cannabigerol (CBG) | 3.80 | 3.02 | 0.10 |
| Cannabigerolic Acid (CBGA) | n.d. | n.d. | n.d. |
| Cannabichromene (CBC) | 0.13 | n.d. | 0.10 |
| Tetrahydrocannabidivarin (THCV) | n.d. | n.d. | 0.02 |
| **Sum Cannabinoids** | **97.03** | **93.88** | **4.84** |

n.d. = not detected


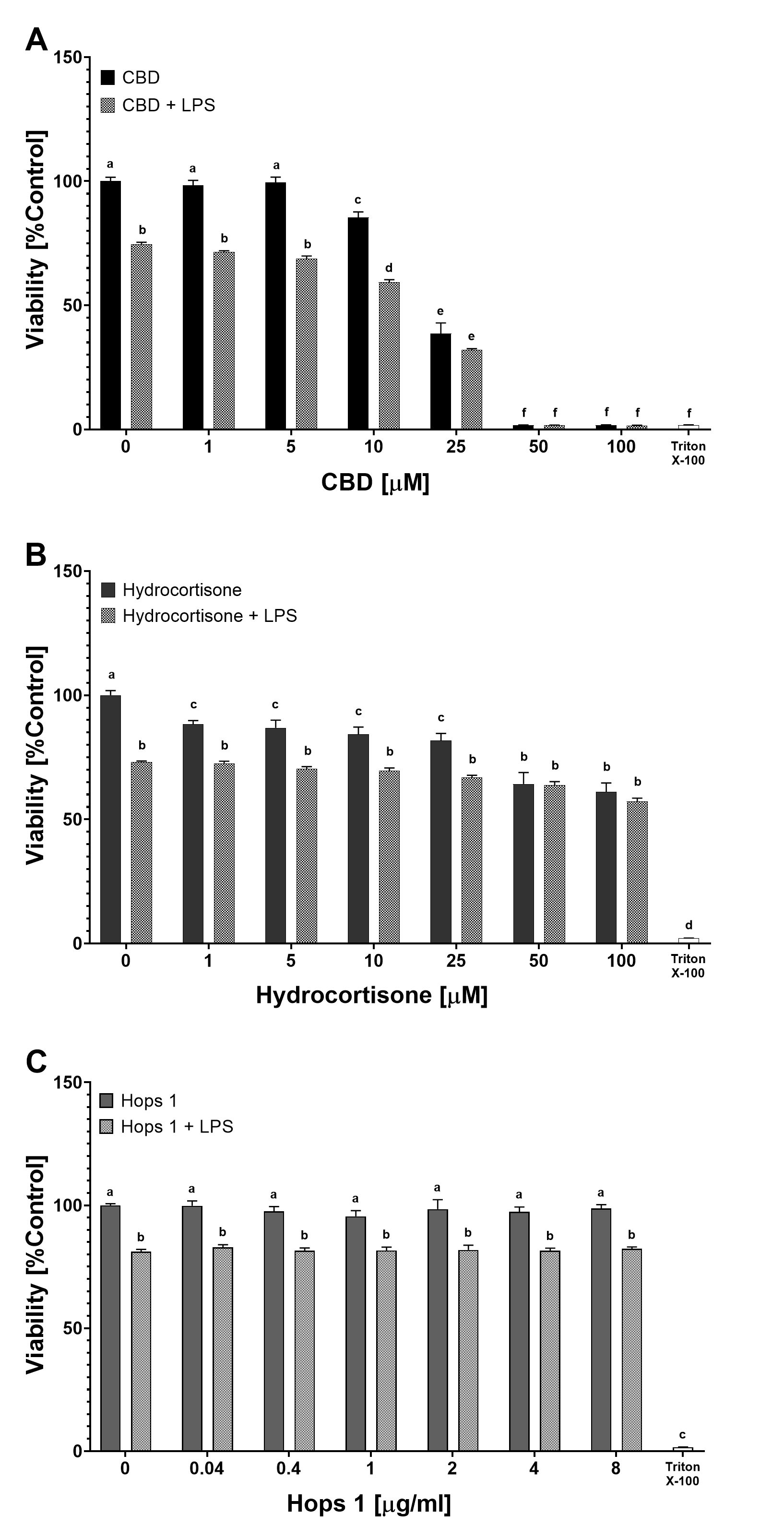


**Figure S1: Toxicity assessment of CBD, hydrocortisone, and Hops 1 extract in RAW 264.7 cells.** Relative cell viability of RAW 264.7 mouse macrophages after incubation with synthetic CBD **(a)**, hydrocortisone **(b)**, or Hops 1 extract **(c)** and optional stimulation with 100 ng/ml LPS. Control: DMEM (-PR, +1 % P/S, + 10 % FBS), 1 %(v/v) DMSO. Incubation with Triton X-100 [0.1 %(v/v)] results in <5% cell viability. Different letters indicate a statistically significant difference between values. All graphs represent data as means ± SEM from at least 3 independent experiments (two-way ANOVA with Tukey’s multiple comparisons post-hoc test).


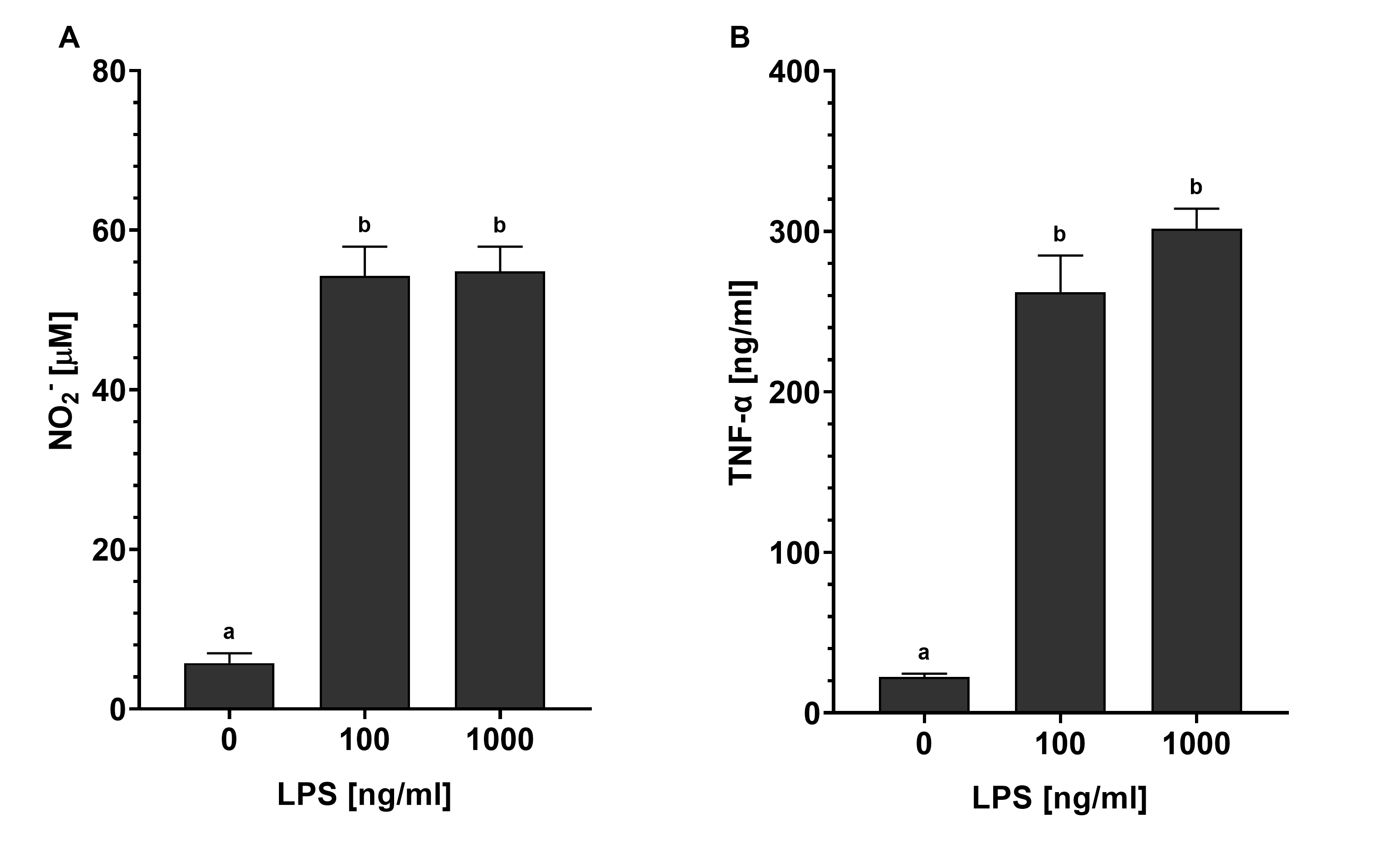


**Figure S2: Amount of nitrite and TNF-**α **in RAW 264.7 supernatants after LPS stimulation.** RAW 264.7 mouse macrophages were grown in DMEM (-PR, +1 % P/S, + 10 % FBS) before LPS stimulation for 24 h. The cell culture supernatants were evaluated for the stable NO• oxidation product nitrite by Griess assay **(a)** and TNF-α was determined by sandwich ELISA **(b)**. LPS stimulation results in the production of nitrite and TNF-α. Different letters indicate a statistically significant difference between values. All graphs represent data as means ± SEM from at least 3 independent experiments (one-way ANOVA with Tukey’s multiple comparisons post-hoc test).


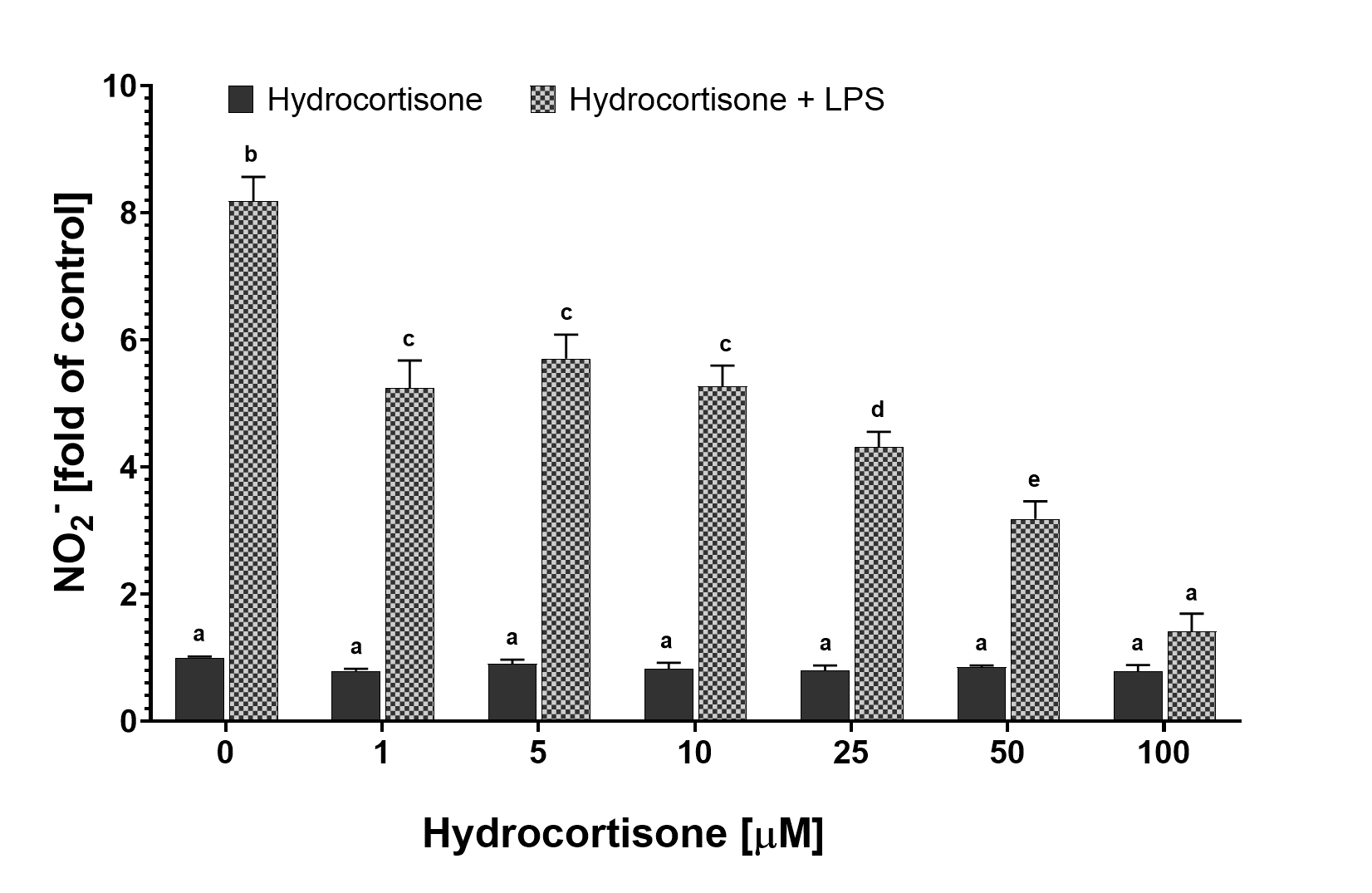


**Figure S3: Influence of hydrocortisone/LPS-treatment on nitric oxide production in RAW 264.7 cells.** Relative amount of the stable NO^·^ oxidation product nitrite in the supernatants of RAW 264.7 mouse macrophages after incubation with hydrocortisone and optional stimulation with 100 ng/ml LPS. Control: DMEM (-PR, +1 % P/S, + 10 % FBS), 1 %(v/v) DMSO. Different letters indicate a statistically significant difference between values. All graphs represent data as means ± SEM from at least 3 independent experiments (two-way ANOVA with Tukey’s multiple comparisons post-hoc test).
